# Phenylalanine promotes alveolar macrophage pyroptosis *via* the activation of CaSR in ARDS

**DOI:** 10.3389/fimmu.2023.1114129

**Published:** 2023-06-12

**Authors:** Yiding Tang, Yue Yu, Ranran Li, Zheying Tao, Li Zhang, Xiaoli Wang, Xiaoling Qi, Yinjiaozhi Li, Tianjiao Meng, Hongping Qu, Mi Zhou, Jing Xu, Jialin Liu

**Affiliations:** ^1^ Department of Critical Care Medicine, Ruijin Hospital, Shanghai Jiao Tong University School of Medicine, Shanghai, China; ^2^ Department of Cardiac Surgery, Ruijin Hospital affiliated to School of Medicine, Shanghai Jiao Tong University, Shanghai, China

**Keywords:** acute respiratory disease syndrome (ARDS), alveolar macrophage (AM), phenylalanine, pyroptosis, CaSR

## Abstract

Acute respiratory distress syndrome (ARDS) is associated with high mortality rates in patients admitted to the intensive care unit (ICU) patients with overwhelming inflammation considered to be an internal cause. The authors’ previous study indicated a potential correlation between phenylalanine levels and lung injury. Phenylalanine induces inflammation by enhancing the innate immune response and the release of pro-inflammatory cytokines. Alveolar macrophages (AMs) can respond to stimuli *via* synthesis and release of inflammatory mediators through pyroptosis, one form of programmed cell death acting through the nucleotide-binging oligomerization domain-like receptors protein 3 (NLRP3) signaling pathway, resulting in the cleavage of caspase-1 and gasdermin D (GSDMD) and the release of interleukin (IL) -1β and IL-18, aggravating lung inflammation and injury in ARDS. In this study, phenylalanine promoted pyroptosis of AMs, which exacerbated lung inflammation and ARDS lethality in mice. Furthermore, phenylalanine initiated the NLRP3 pathway by activating the calcium-sensing receptor (CaSR). These findings uncovered a critical mechanism of action of phenylalanine in the context of ARDS and may be a new treatment target for ARDS.

## Introduction

ARDS, characterized by hypoxemia and bilateral pulmonary edema, and is associated with high mortality rates (34.9%-46.1%) in ICU patients. Overwhelming inflammation is considered to be an internal cause of ARDS (1). Accumulating evidence has revealed the essential roles of metabolites in immune activation and regulation (2). Our previous, which that targeted at the functional metabolites in patients with ARDS indicated a potential correlation between phenylalanine levels and lung injury (3).

Phenylalanine is an essential amino acid that increases in inflammation and the immune response, and has been proposed to predict disease severity in critically ill patients. A recent study reported the accumulation of phenylalanine was associated with inflammatory markers of coronavirus disease 2019(COVID-19) (4). Phenylalanine induces inflammation by enhancing the innate immune response and inducing the release of pro-inflammatory cytokines (5). However, the specific mechanism of action of phenylalanine in the lung inflammation in ARDS remains unclear.

Most airspace leukocytes are AMs. As resident inflammatory cells, they respond to stimuli in the alveolar spaces by synthesizing and releasing inflammatory mediators, which are also vital to inflammation during ARDS. The activation of AMs after exposure to pathogen triggers an inflammatory cascade (6). Pyroptosis is considered to be a mechanism of uncontrolled inflammation, and is a form of programmed cell death, with the cleavage of caspase-1 and GSDMD as well as the release of IL-1β and IL-18 (7). AMs can be activated by pathogen-associated molecular patterns including those of the NLR family. Induced macrophage pyroptosis acts through the NLRP3 inflammasome signaling pathway and aggravates lung inflammation and causes severe lung injury in ARDS (8). Blockade of NLRP3 inflammasome signaling in AMs may suppress pyroptosis and, consequently mitigate lung inflammation and injury in patients with ARDS (9).

In the present study, we demonstrated that phenylalanine promoted pyroptosis in AMs, thus exacerbating lung inflammation and ARDS lethality in mice. We further revealed that phenylalanine initiated NLRP3 pathway by activating CaSR. Thus, our findings may have uncovered a critical mechanism of action of phenylalanine in the context of ARDS lethality.

## Materials and methods

### Patients

This study was approved by Ruijin Hospital Ethics Committee of Shanghai Jiao Tong University School of Medicine. All patients who fulfilled the criteria for ARDS according to the Berlin definition were enrolled in the study (10). Patients < 18 years of age, individuals with any autoimmune diseases, those participating in another clinical trial, and those with other chronic respiratory diseases were excluded.

### Sample acquisition and phenylalanine assessment

Blood samples and bronchoalveolar lavage fluid (BALF) were collected within 9 h of ARDS diagnosis for metabolomic analysis. Collectively, 59 patients were enrolled in this study. Blood samples(2 mL) were collected in heparin tubes and centrifuged at 500×g at 4°C for 5 min, the supernatants were extracted and stored at −80°C untill further use. Phenylalanine levels were assessed using mass spectrometry/high-performance liquid chromatography (Hypersil GOLD HPLC column [ThermoFisher, Waltham, MA, USA] coupled to a QTRAP 6500 [SCIEX, Framingham, MA, USA]). by Mass Spectrometry Platform, National Research Center for Translational Medicine, Ruijin Hospital Affiliated to Shanghai Jiao Tong University (SJTU) School of Medicine, Shanghai, China.

### Animal experiments

Animal experiments were performed using 8–10-week-old C57BL/6 mice purchased from Shanghai Jihui Laboratory Animal Care Co., Ltd. and maintained at in-house facilities under pathogen-free conditions. The animal study was approved by the University Committee for Laboratory Animals and performed in accordance with guidelines of the Shanghai Institutes for Biological Sciences Council on Animal Care. ARDS model animals were established in accordance with a previous report, in which the mice were intratracheally injected with 50 μL bacterial lipopolysaccharide (LPS, Thermo Fisher, #L2630) with the concentration of 6 mg/ml or an equal volume of phosphate-buffered saline (PBS) as sham control (3). ARDS mice were administered with phenylalanine or PBS (25 mg/ml in total volume of 200μl intravenously) 24h before, immediately after and 12h after intratracheal injection of LPS three times. To illustrate the effect of phenylalanine on ARDS lethality, mice were pretreated with phenylalanine or PBS 24 h before the intratracheal injection of LPS, and another dose of phenylalanine or PBS every 24 h until death over the consequent 7 days. To deplete AMs, mice were intratracheally injected with 75 μL of Clodronate Liposomes (CL, Yeason, #40337ES10) 24 h before and soon after the ARDS model was established. Mice in the control group were administered with the same volume of liposomes. BALF and lung tissues were isolated for subsequent experiments 24 h after the final CL injection. To test the role of activated CaSR, another group of mice was administered Calhex231 *via* saline/dimethyl sulfoxide injection intraperitoneally 4 h after phenylalanine administration based on ARDS mice administered phenylalanine.

### Assessment of lung injury

BALF was obtained from the mice *via* tracheal cannulation in a total volume of 1.5 ml PBS. The samples were then placed on ice and directly transferred for further processing. BALF was centrifuged at 500 ×g for 5 minutes at 4°C. The supernatant was tested to determine the concentration of IL-1β, IL-18, and protein concentration using a commercially available kit in accordance with manufacturer’s instructions. Red blood cell lysate was added to the cell precipitate, and the mixture was centrifuged at 500 ×g for 5 min at 4°C after storage for 3 minutes at 4°C. Cells were cultured in RPMI 1640 medium containing fetal bovine serum (FBS) (0.5%) for 2 h and washed with RPMI 1640/0.5% FBS. Lung tissues were prepared for hematoxylin and eosin (H&E) staining.

### Flowcytometry of lung cells

Lung tissue from the mice was crushed into small pieces and digested using 50 µL collagenase I (0.1 mg/mL) (Collagenase, Type 1, Sangon Biotech, Shanghai, China) at 37 °C for 1 h to prepare a single-cell suspension. The following fluorescent antibodies were purchased from Biolegend: APC/Cyanine7 anti-mouse CD45, 103116; PE anti-mouse CD11c, 117307; Brilliant Violet 421 anti-mouse F4/80, 123131; PerCP-Cy™5.5 rat anti-mouse I-A/I-E, 107626; Phycoerythrin/Cy7 anti-mouse/human CD11b, 101216; Brilliant Violet 605 anti-mouse Gr-1, 108439; APC Anti-mouse TCR 118116. Dead/live staining (LIVE/DEAD™ Fixable Aqua Dead Cell Stain Kit) from Invitrogen (Carlsbad, CA, USA) was used to distinguish dead cells and debris.

### Acquisition of bone marrow-derived macrophages and cell culture

Bone marrow cells from 8-week-old C57BL/6 mice were collected by flushing the femurs and tibias and then the cells culturing in α-MEM (Gibco) containing 10% FBS, 100 IU/ml penicillin and 100 mg/ml streptomycin. Culture medium was transferred to another dish on the second day to cultivate non-adherent cells with addition of 10 ng/ml Granulocyte-Macrophage Colony-Stimulating Factor (GM-CSF, # 315-03, Peprotech, USA) at 37°C in a 5% CO2 incubator. The medium was replaced after 3-5 days, and adherent bone marrow-derived macrophages (BMDMs) were used within two passages.

BMDMs were exposed to 100 ng/ml LPS to enhance their proinflammatory phenotype with application of 600 μM Phenylalanine and/or 10 μM Calhex 231 for 4 h. Thirty minutes before the end of the experiment, 2mM ATP was added to the cells. After treatment, the cells were either lysed for Western blotting or collected for flow cytometry to assess pyroptosis.

### Confirmation of pyroptosis

Western blotting and flow cytometry were performed to confirm pyroptosis. The protein concentrations of all samples were estimated using a commercially available BCA kit (Thermo Fischer). Equal amounts of proteins were separated using 10% sodium dodecyl sulfate-polyacrylamide gel electrophoresis and transferred onto a PVDF membrane (Millipore) using a protein transfer system. The membranes were blocked using 5% skim milk powder in TBST buffer for 1 h at room temperature (RT). The blots were then incubated overnight at 4°C with the primary antibodies including mouse anti-rabbit HSP90 (1:2000, Proteintech), mouse anti-rabbit GSDMD (1:1000, Santa Cruz), mouse anti- rabbit N-GSDMD (1:1000, Proteintech), mouse anti-rabbit caspase-1 (1:1000, Santa Cruz), mouse anti-rabbit N-caspase-1 (1:1000, Santa Cruz) and mouse anti-mouse GAPDH (1:1000, Santa Cruz). Subsequently, blots were incubated with anti-mouse or anti-rabbit IgG antibodies (1:5000, Cell signaling) for 1 h at RT. The blots were developed using a commercially available enhanced chemiluminescence detection kit. Each experiment was performed at least twice using cell populations obtained from separate mouse groups. Cells administered different treatments were processed according to manufacturer’s instructions for the annexin V/propidium iodide (PI) assay (CAT#40302ES20, Yeasen, China).

### Measurement of calcium concentration in BMDM

Cells were plated in black 96-well plates at 30,000 cells/well and left to stabilize overnight in a 37°C/5% CO2 incubator. The calcium-regulated intracellular calcium indicator, Fluo-4 AM (Yeasen, 40704ES72) was used to monitor real-time elevations in intracellular calcium following activation or inhibition of the CaSR according to manufacturer’s instructions. Data were acquired using a plate reader at 37°C, with excitation at 494 nm and emission at 516 nm (Synergy Neo, USA).

### Immunofluorescence

Lung tissue slices and BMDMs were isolated as previously described. The slices or cells were fixed using pre-cooled 4% paraformaldehyde for 15 min, washed with PBS, blocked in 3% bovine serum albumin for 1 h, and reacted with rabbit anti-GSDMD (1:100, Bio-Techne China Co. Ltd., China) primary antibody overnight. After washing the samples three times, samples were stained with Alexa Fluor 488 goat anti-rabbit IgG (1:1000, Abcam, USA) for 1 h in the dark. After washing with PBS, the sample slides were incubated with an anti-fluorescence quencher (DAPI) before capping the microscope slides. Slides were observed under a laser-scanning confocal microscope (LSM880, Zeiss, Oberkochen, Germany). Images were analyzed using ZEN software (Zeiss, Germany).

### Statistical analysis

**Figure 1:**
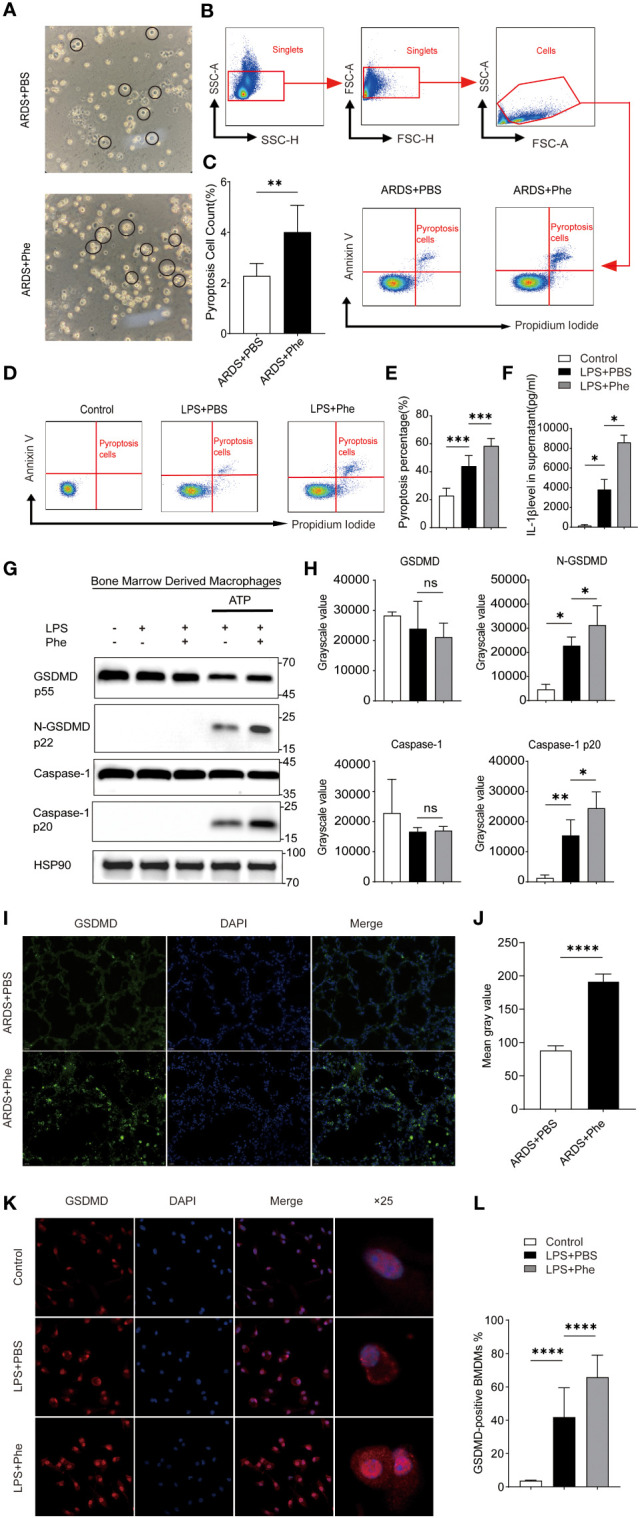
Pyroptosis occurred in AMs of ARDS mice administered phenylalanine and LPS-induced BMDMs treated with phenylananine. **(A)** Two morphological types of AMs in BALF of ARDS mice. **(B)** Cytometry gating workflow of dish-adherent cells in BALF from ARDS mice. **(C)** Pyroptosis (PI+, annexin V+) of dish-adherent cells in BALF from ARDS mice. **(D)** Pyroptosis (PI+, annexin V+) of BMDM cultured in different media. **(E)** Pyroptosis percentage of BMDMs cultured in different media. **(F)** IL-1β level in supernatent of different treatment. **(G)** Western blot of BMDMs cultured in different media. **(H)** Grayscale values for each blot of BMDMs cultured in different media. **(I)** Immunofluorescence detection of GSDMD expression in the lungs of ARDS mice. **(J)** Mean gray value for GSDMD expression in the lungs of ARDS mice. 4**(K)** Immunofluorescence detection of GSDMD expression in BMDM. **(L)** The proportion of GSDMD-positive BMDMs under different treatment. ns means no significance, * means p<0.05, **means p<0.01, ***means p<0.001, ****means p<0.0001.

**Figure 2:**
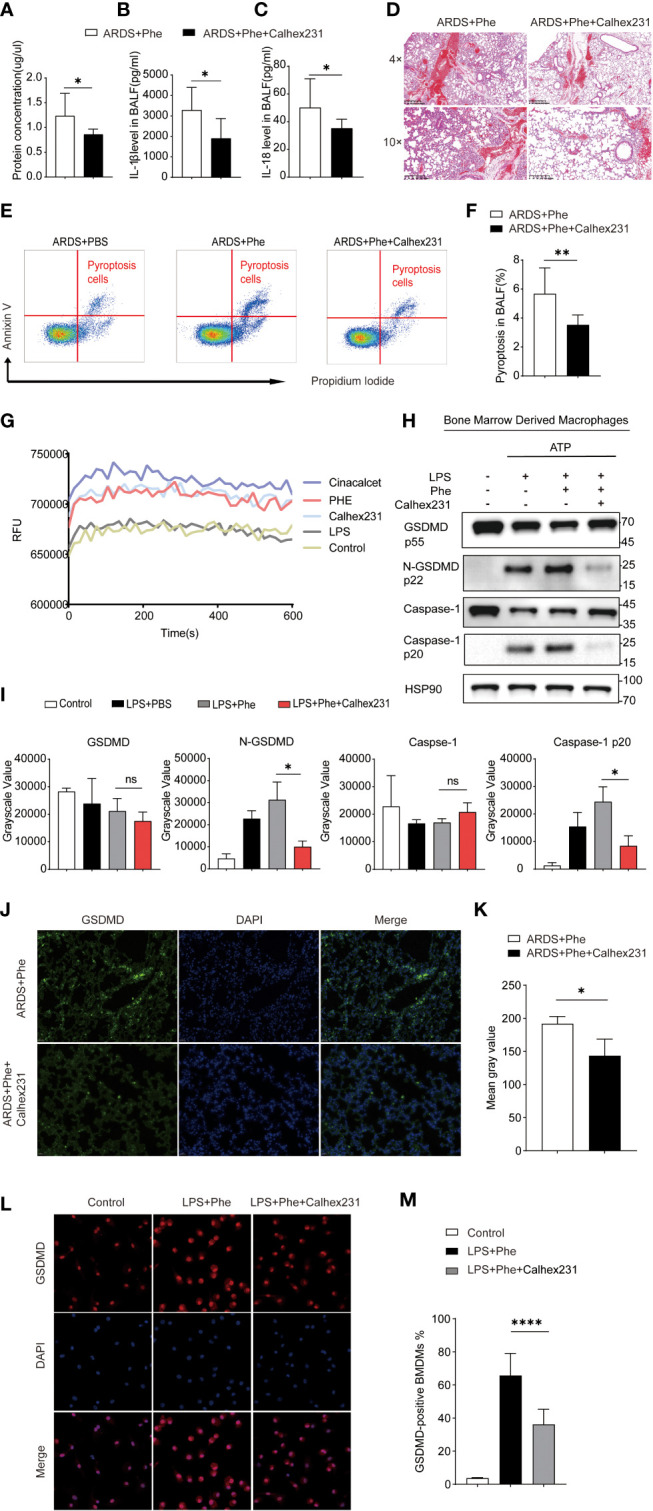
Phenylalanine administration induced pyroptosis *via* CaSR. **(A)** Protein concentration of BALF drawn from two groups of mice. **(B)** IL-1β level in BALF drawn from two groups of mice. **(C)** IL-18 level in BALF drawn from two groups of mice **(D)** Hematoxylin-Eosin staining of two groups of mice. **(E)** Cytometry gating workflow of dish-adherent cells in BALF from two groups of ARDS mice marked with PI and Annexin V. **(F)** Pyroptosis (PI+, annexin V+) proportion of dish-adherent cells in BALF from two groups of ARDS mice. **(G)** Instant relative fluorescence units (RFU) of BMDM treated with different stimulation. **(H)** Western blot of BMDM treated with different stimulation. **(I)** Grayscale values for each blot of BMDMs from different culture. **(J)** Immunofluorescence detection of GSDMD expression in the lungs of ARDS mice with or with out Calhex231. **(K)** Mean gray values for GSDMD expression in the lungs of ARDS mice with or with out Calhex231. **(L)** Immunofluorescence detection of GSDMD-positive BMDMs with or with out Calhex231. **(M)** GSDMD-positive BMDMs percentage with or with out Calhex231. ns means no significance, * means p<0.05, ****means p<0.0001.

**Figure 3:**
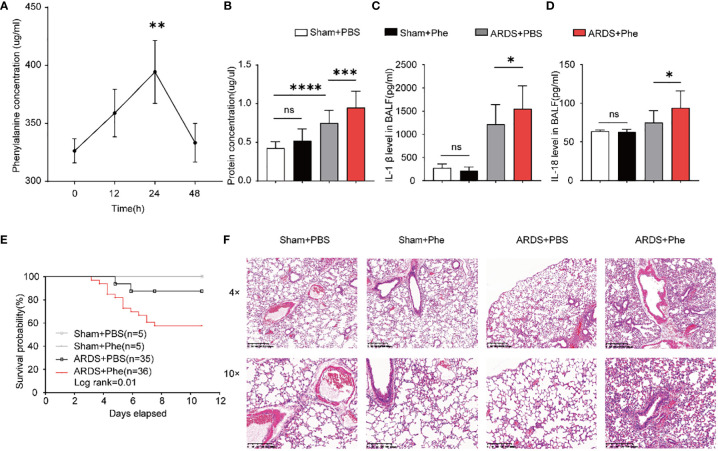
More severe lung injury in phenylalanine-administered ARDS mice. **(A)** The accumulation of phenylalanine in BALF after intravenous injection. **(B)** Protein concentration of BALF drawn from four groups of mice. **(C)** IL-1β level in BALF drawn from four groups of mice. **(D)** IL-18 level in BALF drawn from four groups of mice **(E)** Probability of survival of four groups of mice (collected data of experiments repeated for three times). **(F)** HE staining of four groups of mice. Ns, no significance. **p<0.01. ***p<0.001. ****p<0.0001.

**Figure 4:**
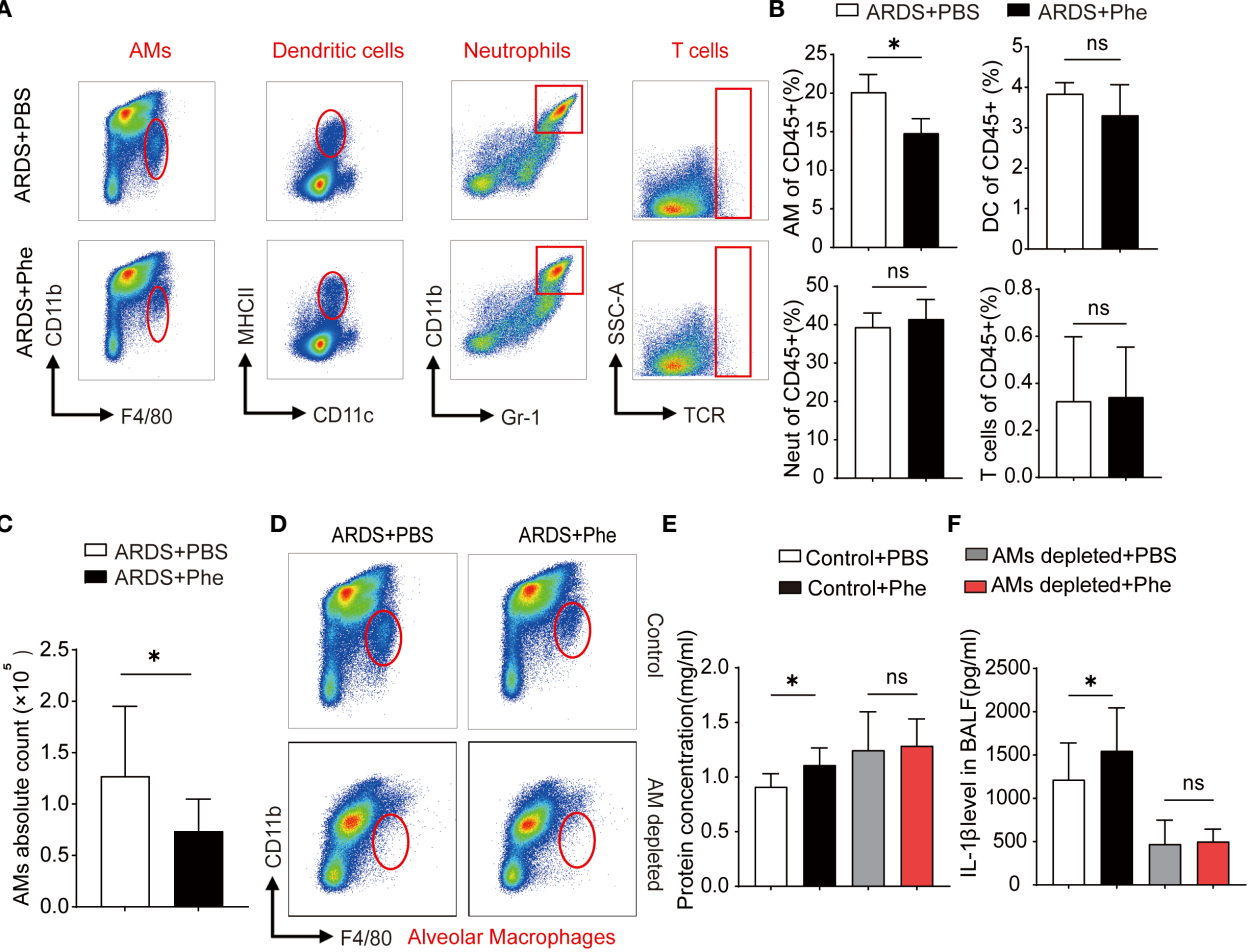
Decreased AMs found in ARDS mice administered Phe. **(A)** Cytometry of four types of immunocytes located in the lungs of ARDS mice administered with PBS or Phe. **(B)** Proportion of related immunocytes in CD45 positive cells. **(C)** Absolute numbers of AMs in ARDS mice administered with PBS or Phe. **(D)** Flow cytometry of AMs in the lung tissue of macrophage-depleted ARDS mice administered with PBS or Phe. **(E)** Protein concentration of BALF drawn from AM-eliminated or control ARDS mice administered PBS or Phe. **(F)** IL-1β level in BALF drawn from four groups of ARDS mice. ns means no significance, * means p<0.05.

Statistical analyses were performed using GraphPad Prism version 9 (GraphPad Inc, San Diego, CA, USA) and SPSS version 24 (IBM Corporation, Armonk, NY, USA). Two-tailed unpaired t-tests, chi-squared tests and Fisher’s exact tests were used for analysis. Receiver operating characteristic (ROC) curve analyses were reported as the area under the ROC curve (AUC). Differences with P < 0.05 were considered to be statistically significant. We repeated cell experiments for more than three times and extract protein for WB. Grayscale value in **Figures 1H**, **2J** collected relative data reading from Image J. Unless specific notes in the figure legend, all the rest plots in **Figures 1**–**4** showed data of one experiment meanwhile every kind of experiment had been repeated for more than three times.

## Results

### Increased serum phenylalanine level was associated with a non-survival outcome in patients with ARDS

**Figure 5:**
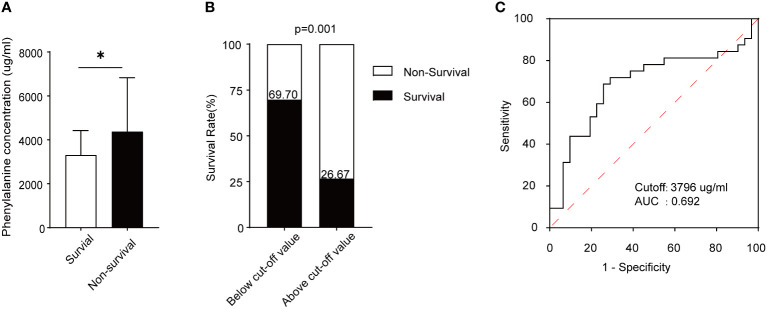
Phenylalanine concentration and its prediction capacity in survival and non-survival ARDS patients. **(A)** Serum phenylalanine concentration in the two groups of patients. **(B)** Survival rate of ARDS patients according to the cut-off value. **(C)** ROC curve and AUC according to the cut-off value. *means p<0.05

From September 2020 to August 2022, 59 patients who fulfilled the Berlin definition criteria for ARDS were enrolled in this study and divided into survival and non-survival groups based on outcomes. The characteristics of each trial participant at the time of plasma collection, including age, sex, ARDS etiology, coexisting conditions, disease severity scores, laboratory results, and outcomes, are summarized in **Table 1**. Appropriate measures were taken to ensure that there was no difference in age or sex between the groups of ARDS patients. As shown, the ratio of partial pressure of oxygen (PaO_2_)/fraction of inspired oxygen (FiO_2_) was significantly higher in survivors than non-survivors, which indicates that the latter experienced impaired lung ventilation and further pulmonary dysfunction. After assessing serum phenylalanine levels in each patient, a significant increase was found in non-survivors compared to survivors (**Figure 5A**). Using ROC analysis, a cut-off value was found that, when serum phenylalanine level reached 3796 μg/ml, the survival of ARDS patients significantly decreased from 69.70% to 26.67 (**Figure 5B**). The ROC curve, according to this cut-off value, was used to assess the ability of phenylalanine level to predict survival rate. (**Figure 5C**).

**Table 1:** Characteristics of enrolled ARDS patients.

Total	59	Survival	Non-survival	P-value
Numbers		27	32	/
Age		64.6 ± 15.4	69.0 ± 12.0	0.23
APACHEII		14.2 ± 5.8	15.9 ± 8.2	0.53
Gender, Female, n(%)		7(26)	16(50)	0.07
Etiology of ARDS, n(%)				
Sepsis		5(23)	5(21)	0.99
Pneumonia		7(32)	14(58)	0.20
Aspiration		1(4)	0	0.49
Trauma		1(5)	1(3)	1.00
Others		11(41)	9(28)	0.58
Coexisting conditions, n(%)				
Diabetes		6(7)	4(13)	0.50
Renal failure		8(30)	6(19)	0.55
Hepatic disease		3(11)	8(25)	0.18
Coronary artery disease		3(11)	3(9)	1.00
Cancer		1(4)	4(13)	0.35
COPD		1(4)	1(3)	1.00
The severity of ARDS, n(%)				
Mild		7(30)	7(25)	0.78
Moderate		12(52)	14(50)	
Severe		4(17)	7(25)	
PaO2/FiO2		208.5 ± 108.0	154.1 ± 74.5	0.04^*^
PEEP, cmH_2_O		8.0 ± 2.8	8.4 ± 3.5	0.68
CRP		127.0 ± 94.3	132.9 ± 92.0	0.64
PCT		10.9 ± 16.5	6.9 ± 16.4	0.26
Cell counts, ×10^9^/L				
Leukocytes		14.4 ± 8.0	13.9 ± 9.0	0.83
Neutrophils		11.8 ± 7.8	15.6 ± 16.8	0.30
Lymphocytes		3.7 ± 5.8	1.1 ± 1.6	0.03^*^
Outcomes of patients				
Ventilator days		18.7 ± 16.3	15.5 ± 14.7	0.48
ICU stay, days		48.1 ± 69.6	39.2 ± 46.6	0.59

Quantitative data are presented as mean ± SD, Qualitative data are presented as number(percentage), P values obtained with Fisher’s two-tailed t test probability using survival (n = 27) and non-survival (n = 32) patients; APACHEII acute physiology and chronic health evaluation; ARDS acute respiratory distress syndrome; COPD chronic obstructive pulmonary disease; PEEP positive end-expiratory pressure; CRP C-reactive protein; PCT procalcitonin; ICU intensive care unit. * P value <0.05.

### Phenylalanine administration aggravated lung inflammation and injury in ARDS mice

Phenylalanine levels were first evaluated in mice challenged with a lethal dose of LPS (25 mg/kg body weight body weight) to confirm the results in samples from patients with ARDS (**Supplementary Figure 1**). To determine whether phenylalanine affected the pathogenesis and prognosis of ARDS, its effect was confirmed in ARDS mice administered three intravenous doses of phenylalanine. Individual BALF samples were obtained from the mice and immediately processed 24h after intratracheal administration of LPS. First, the accumulation of phenylalanine in BALF after intravenous injection was confirmed (**Figure 3A**). The groups of mice exhibited different protein concentrations. Among non-ARDS mice, there was no statistically significant difference regardless of whether phenylalanine was administered. In ARDS mice, the group administered phenylalanine exhibited higher protein levels, indicating more severe lung injury. (**Figure 3B**). Similarly, IL-1β and IL-18 levels also revealed a more serious outcome of phenylalanine-administered ARDS mice compared to control ARDS mice, and the difference disappeared in the sham groups (**Figures 3C, D**). None of the sham mice died during the chronic administration procedure. However, ARDS mice injected with phenylalanine every 12h tended exhibit significantly higher mortality rates than PBS-administered ARDS mice (**Figure 3E**). H&E staining of lung tissues revealed more diffusion in phenylalanine-injected ARDS mice than in PBS-injected ARDS mice at both 4× and 10× magnification. No significant differences were observed between the two sham groups (**Figure 3F**).

### Phenylalanine administration reduced AMs in ARDS mice

After demonstrating severe lung injury in phenylalanine-administered ARDS mice, more studies involving the two groups were performed. First, the composition of intrapulmonary immunocytes in ARDS mice was analyzed. Using these methods, intrapulmonary cells were isolated and immunocytes were divided into dendritic cells (DCs), neutrophils (Neuts), AMs, and T cells (**Supplementary Figure 2**). Comparison of the immunocyte composition of the two groups of ARDS mice revealed more intrapulmonary AMs in PBS-administered ARDS mice than in phenylalanine-administered ARDS mice, whereas no significant difference was observed in DCs, Neuts, and T cells (**Figure 4A**). Statistical analysis confirmed a significantly lower percentage of intrapulmonary AMs in the phenylalanine-treated ARDS mice than in the PBS-treated ARDS mic (**Figure 4B**). To further support this finding, the absolute number of AMs was calculated (**Figure 4C**), which suggested that the difference in AMs resulted in more severe lung injury in phenylalanine-treated ARDS mice. Therefore, intrapulmonary AMs in ARDS mice were further depleted to explore whether there was a difference in the severity of lung injury without AMs (**Figure 4D**). Results revealed that protein concentration and IL-1β level in BALF after the depletion of AMs demonstrated no significant difference between the two groups (**Figures 4E, F**).

### Phenylalanine administration induced pyroptosis in activated AMs

After confirming that AMs may be the key cells that lead to phenotypic differences between ARDS mice with different levels of phenylalanine, BALF was extracted from the two groups of ARDS mice and cells were cultured to divide them into AMs (adherent cells) and Neuts (suspended cells). It was clear that the morphology of AMs can be roughly divided into two types: steady state, with round and complete cell membrane and opaque interior; and those in a state of pyroptosis with cell swelling and expansion and many bubble-like protrusions (**Figure 1A**). The PBS-treated ARDS mice exhibited more intact and opaque AMs, whereas the phenylalanine-treated ARDS mice exhibited more pyroptotic cells. Flow cytometry analysis of these adherent cells stained with PI and annexin V revealed that the proportion of pyroptotic cells (double-positive) was significantly higher in phenylalanine-treated ARDS mice (**Figures 1B, C**). Similar *in vitro* tests were performed on BMDMs from the same source as AMs. The proportion of pyroptotic cells was significantly higher in the medium supplemented with phenylalanine (**Figures 1D, E**). IL-1β level of supernatant also exhibited a significant increase when cells were treated with phenylalanine (**Figure 1F**). In addition, Western blotting demonstrated significantly upregulated expression of cleaved-GSDMD and cleaved-caspase-1 in BMDMs treated with phenylalanine (**Figures 1G, H**). Immunofluorescence detection detection increased expression of GSDMD in the lungs of phenylalanine-treated ARDS mice (**Figure 1I**), which was confirmed by quantification of the mean gray value (**Figure 1J**). When detecting GSDMD in cells, GSDMD expression was obviously promoted in BMDMs with LPS and phenylalanine (**Figure 1K**). The proportion of GSDMD-positive BMDM expressing phenylalanine was significantly higher (**Figure 1L**). Collectively, these results demonstrated that pyroptosis occurred and was promoted in both AMs of ARDS mice administered phenylalanine and in BMDMs treated with phenylalanine.

### Phenylalanine administration induced pyroptosis *via* the calcium-sensitive receptor

Phenylalanine is a positive allosteric regulator of the CaSR, which is involved in various cellular interactions (11). Moreover, GSDMD and caspase-1 are downstream effector molecules present in their cleaved forms when activated. Therefore, the authors hypothesized that phenylalanine may promote the cleavage of GSDMD and caspase-1 by activating the CaSR to induce pyroptosis. To verify the role of the CaSR, its specific inhibitor Calhex231 was applied to ARDS mice to observe relief of lung injury and inflammation. The protein concentrations of IL-1β and IL-18 in BALF decreased (**Figures 2A–C**). H&E staining of the lung tissue exhibited less diffusion in Calhex231-administered ARDS mice (**Figure 2D**). In addition, a significantly lower proportion of pyroptosis in BALF cells from mice administered Calhex231 was found (**Figures 2E, F**). By determining calcium influx into cells, measured in relative fluorescence units (RFU), cells treated with phenylalanine exhibited significantly higher calcium influx, and CaSR-specific stimulants further promoted calcium influx into cells (**Figure 2G**). In addition, Western blot analysis of BMDMs in different media revealed that the expression of cleaved-GSDMD and cleaved-caspase-1 was significantly downregulated when the medium was supplemented with Calhex231 (**Figures 2H, I**). When phenylalanine-administered ARDS mice were applied with Calhex231, the expression of GSDMD in the lungs was significantly downregulated (**Figures 2J, K**). GSDMD-positive BMDMs drastically decreased in the presence of Calhex231. Accordingly, fewer GSDMD-positive BMDMs were detected in Calhex231-administered cells in comparison to only Phe-treated BMDMs (**Figures 2L, M**). We further assessed the role of caspases and NLRP3 with their inhibitors Z-Val-Ala-Asp-FMK (**Supplementary Figure 3**) and MCC950 (**Supplementary Figure 4**). This indicates that pyroptosis was associated with the CASR *via* activation of NLRP3 and Caspase-1.

## Discussion

To our knowledge, this is the first study to reveal a novel role for phenylalanine in mediating ARDS *via* induction of pyroptosis in AMs. We first observed that increased phenylalanine levels in ARDS patients was associated with mortality. In mice with ARDS, phenylalanine administration has been shown to be closely associated with increased AM pyroptosis, along with elevated levels of IL-1β and IL-18. We performed *in vivo* and *in vitro* investigations to further confirm that phenylalanine was responsible for the increase in AM pyroptosis *via* the activation of the CaSR-initiated NLRP3 inflammasome pathway. CaSR inhibition significantly alleviates LPS-induced inflammation and decreases the frequency of AM pyroptosis. For the first time, we have elucidated a complete pathway of how elevated phenylalanine levels trigger AM pyroptosis, resulting in inflammation when exposed to higher levels of phenylalanine and leading to ARDS lethality, which expands the current understanding of the role of small-molecule metabolites in pulmonary inflammation. These findings may provide new treatment targets for patients with ARDS.

Metabolites have been proposed as novel biomarkers reflecting the severity of ARDS owing to their quick response to the influence of external factors (12). In fact, metabolites are not only downstream products but also active drivers of biological processes serving as molecular signals (13). Our study highlights the association between elevated phenylalanine levels and poor outcomes in patients with ARDS. In our study, the administration of phenylalanine aggravated lung inflammation, reflected by elevated levels of IL-1β and IL-18 in BALF. These results implicate phenylalanine as a “bioactive metabolite” in physiological reactions in ARDS. Phenylalanine is an essential amino acid (14) that is absorbed into plasma and is mainly converted to tyrosine in the liver (15). The lungs do not directly use phenylalanine; however, consistent with previous research by Tan et al. (16), our study found that the lungs take up phenylalanine, which was demonstrated by the accumulation of phenylalanine in BALF after intravenous injection (**Supplementary Figure 1**). These results provided basic evidence that phenylalanine directly interacts with lung cells.

AMs, the major immunocytes regulating lung inflammation and acute lung injury (ALI), respond to invasive stimuli *via* pyroptosis (17), which is a caspase-1-dependent death pathway. Activated caspase-1 cleaves GSDMD to generate mature GSDMD that induces plasma membrane pore formation, resulting in cell swelling, plasma membrane rupture, and the release of pro-inflammatory cytokines such as IL-1β and IL-18 (18). It has been reported that excessive pyroptosis can cause partial or systemic inflammation and even lead to lethality (19). Zhu et al. found that AM pyroptosis was elevated when the lung structure was disrupted by cigarette smoke (20). In addition, Wu et al. reported that caspase-1 was activated in LPS-induced ALI, thereby facilitating AMs pyroptosis (21). In our study, phenylalanine administration promoted pyroptosis in AMs, which in turn is believed to induce severe lung injury. Depletion of AMs eliminated the adverse outcomes caused by phenylalanine. Therefore, AM pyroptosis resulting from elevated phenylalanine levels is the main trigger of severe lung injury in ARDS.

The NLRP3 inflammasome is a critical component of the innate immune system (22). The activation of the NLRP3 pyrolytic pathway requires two signals simultaneously. The first includes pathogenic agents, such as LPS, or cytokines, and the former includes lysosomes and ATP. The first signal increases the activation of caspase-1 and GSDMD in the pyroptotic pathway, whereas the second signal is a key step in the cleavage and assembly of inflammasomes (23). Recent studies have shown that elevated calcium (Ca^2+^) concentrations can promote the combination of NLRP3 and ASC proteins to form activated inflammasomes, thereby ensuring the conduction of downstream pathways (24). Our results suggest that phenylalanine upregulates intracellular Ca^2+^ concentrations by activating CaSR, a regulator of calcium homeostasis, and its inhibition can downregulate pyroptosis. Phenylalanine does not affect NLRP3 expression. Among all aromatic L-amino acids, phenylalanine is most likely to bind and activate the CaSR (25). Therefore, our results indicate that phenylalanine serves as a secondary signal that may bind to the CaSR and recruit Ca^2+^, accelerating the assembly of NLRP3 and ASC, and finally leading to the activation of NLRP3 mediated pyroptosis. CaSR, a member of the aromatic L-amino acid receptor family, is a G-protein-coupled receptor distributed in the lung tissue and expressed in macrophages (26). It is widely associated with inflammation in diverse diseases involving the cardiovascular system (27), such as vascular calcification, atherosclerosis, myocardial infarction, hypertension, obesity (28) and the respiratory system, such as asthma (29) and acute lung injury (30). Results of our study demonstrated that CaSR inhibition alleviated phenylalanine-induced lung injury, inflammation, and AM pyroptosis, further confirming that phenylalanine plays a role *via* the CaSR.

We have limited clinical samples and there may still exist other minor pathways to pyroptosis in above situation. Nevertheless, we demonstrated a novel mechanism by which elevated phenylalanine levels promote inflammation *via* the CaSR and aggravates ARDS through the induction of AM pyroptosis, which may be a new target for treating ARDS.

## Data availability statement

The raw data supporting the conclusions of this article will be made available by the authors, without undue reservation.

## Ethics statement

The studies involving human participants were reviewed and approved by Ruijin Hospital Ethics Committee of Shanghai Jiao Tong University School of Medicine. The patients/participants provided their written informed consent to participate in this study. The animal study was reviewed and approved by Shanghai Institutes for Biological Sciences Council on Animal Care.

## Author contributions

YT designed research studies, conducted experiments, acquired clinical data, analyzed data, wrote the manuscript. YY collected clinical samples. RL designed research studies. ZT analyzed data. LZ conducted experiments. XW, XQ, YL, TM and HQ collected clinical samples. MZ provided reagents. JX designed research studies and wrote the manuscript. JL designed research studies and revised the manuscript. YT is in the first place of first author and YY is in the second place of the co-first author. JL is in the first place of respondence, JX is in the second place of respondence and MZ is in the third place of respondence. All authors contributed to the article and approved the submitted version.
