# Supplementary material for: Phenylalanine promotes alveolar macrophage pyroptosis via the activation of CaSR in ARDS
Source: Front Immunol. 2023 Jun 12;14:1114129. doi: 10.3389/fimmu.2023.1114129 (PMC10291621; doi:10.3389/fimmu.2023.1114129)
Supplement: Supplementary file 1 [file Presentation_1.pdf]

Supplementary Figures

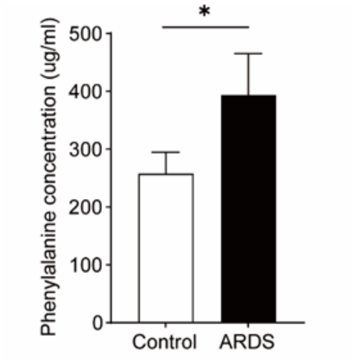

Supplementary Fig.1 Levels of phenylalanine in serum of mice challenged with PBS vs lethal dose of LPS (25mgLPS per kilogram body weight)

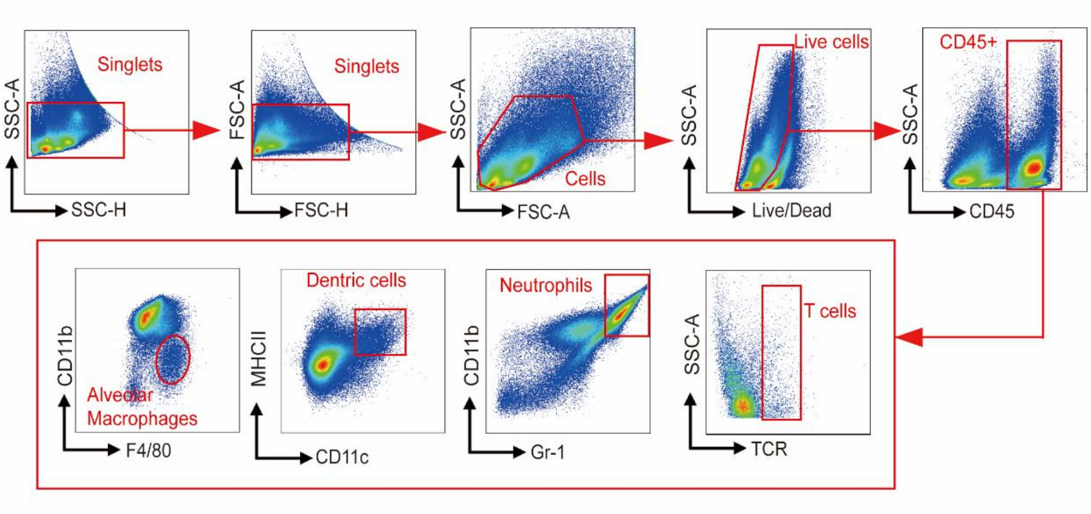

Supplementary Fig.2 Cytometry gating workflow of dendritic cells (DC), neutrophils (Neut), alveolar macrophages (AMs) and T cells in lung tissues of ARDS mice administrated with PBS or phenylalanine.

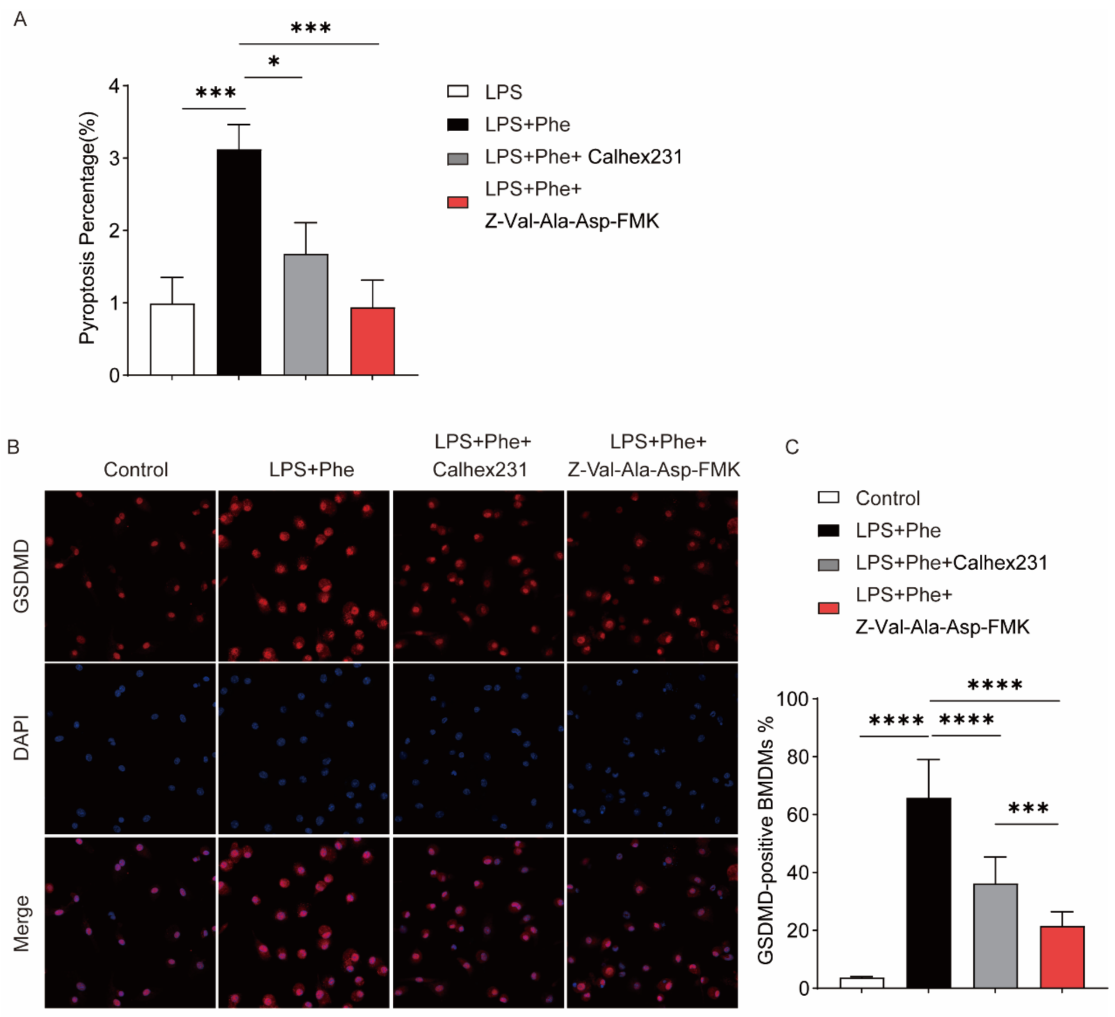

Supplementary Fig.3 Pyroptosis detection with applications of pan-caspase inhibitor Z-Val-Ala-Asp-FMK (50mM, Selleck). A) Pyroptosis (PI+, Annixin V+) of BMDM cultured in different media. B) Immunofluorescence detection of GSDMD expression of GSDMD-positive BMDM. C) GSDMD-positive cell percentage of each treatment.

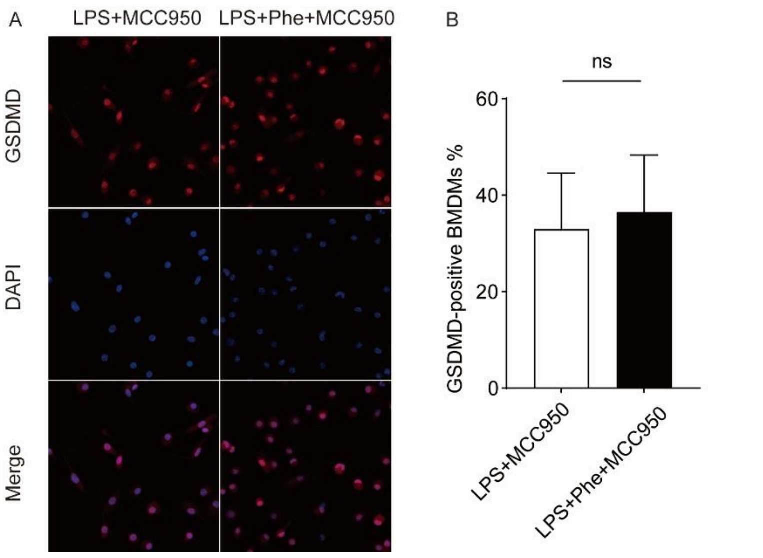

Supplementary Fig.4 Pyroptosis detection with applications of specific NLRP3 inhibitor MCC950 (10mM, Selleck). A) Immunofluorescence detection of GSDMD expression of GSDMD-positive BMDM. B) GSDMD-positive cell percentage of each treatment.
